# Overview on antibacterial metabolites from terrestrial *Aspergillus* spp

**DOI:** 10.1080/21501203.2019.1604576

**Published:** 2019-04-22

**Authors:** Abdulkawi Ali Al-Fakih, Wael Qasem Abdulgabbar Almaqtri

**Affiliations:** a Department of Medical Microbiology, Faculty of Science, Ibb University, Ibb, Yemen; b Department of Chemistry, Faculty of Science, Ibb University, Ibb, Yemen

**Keywords:** Aspergillus, terrestrial, antibacterial, extracts, metabolites

## Abstract

Medicines developed from natural sources are a frequent target for the research and discovery of antimicrobial compounds. Discovering of penicillin in 1928 was a motive to explore of nature as a source of new antimicrobial agents. Fungi produce a diverse range of bioactive metabolites, making them rich source of different types of medicines. The purpose of this paper was to review studies on antibacterials from terrestrial *Aspergillus* published exclusively during 1942–2018, with emphasis on their antibacterial activities, structures, and mechanisms of action if present. According to the results from different studies in the world, large number of compounds and extracts showed different activities against different bacterial species, including Gram-positive and Gram-negative bacteria. The most prominent result was that of the compound CJ-17,665, isolated from *A. ochraceus*, showing good activity against multi-drug resistant *Staphylococcus aureus*, which is well-recognised to be one of the most important current public health problem. These findings may motivate scientists to undertake a project that may result in the development of novel antibacterial drugs from terrestrial-derived *Aspergillus* spp., although further toxicity assays (*in vivo*) must be performed before their application.

## 1. Introduction

Fungi represent one of humanity’s oldest domesticated organisms and are responsible for the production of some of industrial (e.g. alcohol), nutritious (e.g. bread), and medically useful (e.g. penicillin) products (Kavanagh 2011). From the beginning until now, the humankind has always been faced with a problem of spreading of bacterial infections. The kingdom fungi (Mycetae) is well-known as a valuable source of diverse bioactive compounds of therapeutic importance since the discovery of penicillin in 1928 by Alexander Fleming (Fleming 1929; Clutterbuck et al. 1932) from *Penicillium notatum*. The treatment of bacterial infections is increasingly complicated by the ability of bacteria to develop resistance to antibacterial agents (Levy and Marshall 2004). Therefore, an urgent need to exploit new classes of antibacterial agents with a novel mechanism of action is required. The increasing need for new antibacterial agents able to control emerging diseases or resistant strains of bacteria inspired a number of research groups to explore the terrestrial and marine environments for new antibacterial compounds (Ng et al. 2015). Filamentous fungi represent an important group of microorganisms known to synthesise a vast diversity of bioactive molecules that are traditionally termed secondary metabolites or natural products (Calvo et al. 2002; Horgan and Murphy 2011; Jansen et al. 2013; Bills and Stadler 2014). Secondary metabolites are biologically active organic compounds that are not required for normal growth, development, or reproduction, but provide a competitive advantage to the producing organism. Secondary metabolites are used as medicines, flavourings, pigments, and recreational drugs (Craney et al. 2013). The discovery of penicillin was a real revolution in medicine and the research interest to explore new antibacterial agents from microorganisms is continued. Many intensive studies, mainly on terrestrial-derived and marine-derived fungi, displayed that fungi are a rich source of unique bioactive substances. A large number of antibacterial metabolites are identified in many species of fungi throughout the world and some have been approved as drugs, such as cephalosporins and fusidic acid (Newton and Abraham 1955; Wo et al. 1962). Most fungi-derived pharmaceuticals have been sourced from Ascomyceteous fungi, such as *Aspergillus, Penicillium*, and so on, whereas perhaps fewer pharmaceuticals, certainly in an industrial context, have been derived from Basidiomycetous fungi and others (Bala et al. 2011). However, more than 30% of isolated metabolites from fungi are from *Aspergillus* and *Penicillium* (Bérdy 2005).

*Aspergillus* species were the most diverse and common fungal species found in the environment. One explanation for the high number of compounds reported from this genus is that *Aspergillus* spp. are salt tolerant, fast growing species, and are easily obtained from many substrates (Bugni and Ireland 2004). Secondary metabolites isolated from species of the genus *Aspergillus* have continually attracted the interest of pharmacologists due to their broad array of biological activities and their structural diversity (Cai et al. 2011; Wang et al. 2017). *Aspergillus* have proven to be a prolific source of secondary metabolites with interesting biological activities, including antibacterial activity (Perrone et al. 2007; Xu et al. 2015). The aim of this review is to present the antibacterial metabolites or extracts described from terrestrial *Aspergillus* spp., which may have pharmaceutical importance as antibacterial agents. In this review, it is noticed that antibacterial compounds were presented in chronological order. In addition, it is worth mentioning that we have collected all relevant information as far as we can.

## 2. Assessment techniques of antibacterial activity of fungal metabolites

To assess the antibacterial activity of fungal extracts or pure compounds, several bioassays are well known and commonly used, such as agar disc diffusion method, agar well diffusion method, microdilution method, and a method with incorporation of the extract in the culture medium and determination of bacterial colonies. Thus, the results of antibacterial activity are expressed in different units. In agar disc diffusion method, the extract is incorporated into discs at different concentrations. The clear or inhibition zone developed around each disc was measured and the antibacterial activity is represented by inhibition zone diameter (IZD) (CLSI 2012). The principle of the agar well diffusion method is the same as that of the agar disc diffusion method, but the extract, in fixed volumes, is placed in wells rather than discs (Valgas et al. 2007). Microdilution method comprises microdilutions of the extract in liquid medium using microplates to determine the values of minimal inhibitory concentration (MIC), minimum bactericidal concentration (MBC), which also known as the minimum lethal concentration (MLC), or a concentration that inhibit 50% of the bacterial growth (IC_50_) (Soothill et al. 1992). With respect to the fourth method, the extract is incorporated in the culture medium and the resultant colony forming units (CFU) are determined. Other bioassays, such as flow cytofluorometric and bioluminescent methods are not widely used because they require specified equipment and further evaluation for reproducibility and standardization (Balouiri et al. 2016). For many details, the above assessment techniques were reviewed by Balouiri et al. (2016).

## 3. Aspergillus

*Aspergillus* (Trichocomaceae) is a genus of ascomycetous fungi that grows in various environments. It has often been isolated from various environments, such as soil (Furtado et al. 2002; El-Aasar 2006; Cai et al. 2011; Amina et al. 2017; Hassan and Bakhiet 2017), various host plant tissues, as endophytic fungi (Kusari et al. 2009; Li et al. 2012; Monggoot et al. 2018), and aquatic environments (Nguyen et al. 2007; Qiao et al. 2010; Fukuda et al. 2014; Li et al. 2018). *Aspergillus* represents a large diverse genus, containing about 180 filamentous fungal species of substantial pharmaceutical and commercial values (Lubertozzi and Keasling 2009; Ibrahim et al. 2015a). *Aspergillus* is defined as a group of conidial fungi – that is, fungi in an asexual state. Some of them, however, are known to have a teleomorph (sexual state) in the Ascomycota, so with DNA evidence forthcoming, members of the genus *Aspergillus* can be considered members of the Ascomycota (Peterson 2008). *Aspergillus* is one of the major contributors to the secondary metabolites of fungal origin (Ibrahim et al. 2015b). It is well-known for its production of mycotoxins, such as aflatoxin, ochratoxin, gliotoxin, fumagillin, helvolic acid (fumigacin), fumitremorgin A, and asphemolysin (Wilson 1966; Bhetariya et al. 2011). These mycotoxins are capable of causing mycotoxicoses in animals and humans. In contrast, *Aspergillus* is currently expanding its application in various fields of medicine and pharmaceuticals. Many compounds, with antifungals, e.g., 3′-(3-Methylbutyl)-butyrolactone II (Cazar et al. 2005), antivirals, e.g., aspergillipeptides and asteltoxins (Tian et al. 2015; Ma et al. 2017a), antiprotozoal, e.g., (22*E*,24*R*)-stigmasta-5,7,22-trien-3-β-ol and stigmast-4-ene-3-one (Ibrahim et al. 2015b), antioxidant, e.g., 2-hydroxycircumdatin C (Cui et al. 2009), antidiabetic, e.g., isoaspulvinone E and aspulvinone E (Dewi et al. 2015), anticancer, e.g., [4-(2-methoxyphenyl)-1-piperazinyl](1-methyl-1H-indol-3-yl)-methanone (He et al. 2012), lipases (Yadav et al. 2000), α-amylases (Saranraj and Stella 2013), probiotics (Lee et al. 2006), and statins (Alberts et al. 1980) are all derived from *Aspergillus*.

### 3.1. Antibacterial metabolites from terrestrial Aspergillus spp

Many species of *Aspergillus* are active producers of many products of industrial and pharmaceutical importance (Mehta et al. 2017; Wang et al. 2015). Recently, a growing number of *Aspergillus* spp. fungi, have been reported to produce novel bioactive compounds, such as butenolide derivatives as anti-inflammatory agents (Liu et al. 2018), aurasperone H as anticancer (Li et al. 2016), asperchondols A and B as antibacterials (Liu et al. 2017), aspergivones A and B with anticancer and antibacterial activities (Ma et al. 2017b), and aspochalasin B and D as antibacterials (Ratnaweera et al. 2016). In this section, we discuss bioactive metabolites of *Aspergillus* spp., isolated from terrestrial environments, that shown antibacterial activities and could provide further cues for clinical trials. Table 1 summarises antibacterial findings reported on the preclinical antibacterial activity of the compounds and extracts from *Aspergillus* spp.

**Table 1.** Antibacterials derived from terrestrial *Aspergillus* spp.

Fungus	Antibacterial(s)	Susceptible bacteria (MIC, IZD, or IC_50_)	Reference
*A. fumigatus*	Helvolic acid	Gram-positive bacteria	(Chain et al. 1943)
*A. giganteus* (Wehm)	Claviformin (patulin)	Gram-positive and Gram-negative bacteria	(Florey et al. 1944)
*A. fumigatus*	Aspergillin	*Mycobacterium tuberculosis*	(Soltys 1944)
*A. niger* (YWA), *A. nidulans, A. oryzae* (TP), and *A. flavipes*	Culture filtrate	Gram-positive and Gram-negative bacteria	(Foster and Karow 1945)
*Aspergillus* spp.	Culture filtrate	*Bacterium coli, S. aureus*, and *Pseudomonas pyocyanea*	(Wilkins and Harris 1945)
*Aspergillus* spp.	Culture filtrate	*S. aureus* and *Salmonella typhi*	(Brian and Hemming 1947)
*A. fumigatus*	Fumagillin	Gram-positive and Gram-negative bacteria	(Hanson and Eble 1949)
*A. caespitosus*	5,6-dihydro-5(S)-acetoxy-6(S)-1,2-trans-epoxypropyl)-2H-pyran-2-one, 5,6-dihydro-5(S)-acetoxy-6(S)-(1,2-trans propenyl)-2H-pyran-2-one, and 5,6-dihydro-5(R)-acetoxy-6(S)-(1,2-trans-epoxy-propyl)-H-pyran-2-one	*Salmonella paratyphi* A (MIC = 50, 50, and 100 μg ml^−1^, respectively)	(Mizuba et al. 1975)
*A. terreus* var. *aureus*	Dihydrogeodin	*Bacillus subtilis* (IFO-3513) (MIC = 6.25 μg ml^−1^)	(Inamori et al. 1983)
*A. quadrilineatus*	Petroleum ether extract of mycelia	*S. aureus* and *B. Subtilis* (IZDs= 18 and 20 mm, respectively)	(Irobi et al. 2000)
*A. ochraceus* (CL41582)	CJ-17,665	*S. aureus* (MDRSA), *S. pyogenes*, and *E. faecalis* (MIC = 12.5, 12.5, and 25 μg ml^−1^, respectively)	(Sugie et al. 2001)
*A. fumigatus*	Chloroform extract of mycelia	*S. aureus* and *M. luteus* (IZDs = 20.67 and 39.33 mm, respectively)	(Furtado et al. 2002)
*A. niger* (IFB-E003)	Rubrofusarin B, fonsecinone A, asperpyrone B, and aurasperone A	*B. subtilis, E. coli*, and *P. fluorescence* (MICs = 1.9-31.2 μg ml^−1^)	(Song et al. 2004)
*Aspergillus* sp. (CY 725)	Helvolic acid, monomethylsulochrin, ergosterol, and 3β-hydroxy-5α,8α-epidioxy-ergosta-6,22-diene	*Helicobacter pylori*	(Li et al. 2005)
*Aspergillus* spp.	Ethyl acetate extract of mycelia	Gram-positive and Gram-negative bacteria (IZDs = 0-15.2 mm)	(Maria et al. 2005)
*A. terreus* var. *terreus*	Terreic acid and Butyrolactone I	*Erwinia carotovora* (IC_50_ = 5.1 and 12.5 μg ml^−1^, by terreic acid and butyrolactone I, respectively), *B. subtilis* and *Micrococcus luteus* (IZDs = 35 and 8 mm, respectively, by terreic acid), and *Bacillus brevis, B. subtilis*, and *Enterobacter dissolvens* (IZDs = of 30, 21, and 17 mm, respectively, by butyrolactone I)	(Cazar et al. 2005)
*A. flavus, A. glaucus, A. oryzae, A. parasiticus*, and *A. tamirii*	Kojic acid	Gram-positive and Gram-negative bacteria (MICs = 176–285 μg ml^−1^)	(El-Aasar 2006)
*A. niger* (FKI-2342)	Tesyuic acid C	*B. subtilis* (IZD= 10 mm)	(Hasegawa et al. 2007)
*A. awamori* (F12)	Emodin	*S. aureus* and *B. subtilis* (MICs = 16 and 32 μg ml^−1^, respectively)	(Chang et al. 2010).
*Aspergillus* sp.	Ethyl acetate extract of mycelia and culture filtrate	Mycelial extract: (*E. coli, K. pneumoniae, S. aureus*, and *P. aeruginosa*; IZDs = 8.6–12 mm) Culture broth: (*P. aeruginosa* and *K. pneumoniae*; IZDs = 13 and 15 mm)	(Prabavathy and Nachiyar 2012).
*A. fumigatus*	Ethyl acetate extract of mycelia	*B. subtilis, E. coli, K. pneumoniae, Shigella flexneri*, and *S. aureus* (IZDs = 16, 18, 20, 11, and 16 mm, respectively)	(Ruma et al. 2013)
*Aspergillus* sp. (EJC08)	Fumigaclavine C and pseurotin A	Fumigaclavine C: (*B. subtilis, E. coli, P. aeruginosa*, and *S. aureus* (MICs = 7.81, 62.5, 31.25, and 15.62 μg ml^−1^, respectively) Pseurotin A: (*B. subtilis, E. coli, P. aeruginosa*, and *S. aureus* (MICs = 15.6, 31.3, 31.3, and 15.6 μg ml^−1^, respectively)	(Pinheiro et al. 2013)
*A. niger*	Culture filtrate	*P. aeruginosa, S. aureus, S. epidermidis*, and *Bacillus* sp. (IZDs = 15, 25, 30, and 32 mm, respectively)	(Al-Shaibani et al. 2013)
*Aspergillus* sp.	Ethyl acetate extract of mycelia	Gram-positive and Gram-negative bacteria (IZDs = 0–21.7 mm)	(Sadrati et al. 2013)
*Aspergillus* sp. (KJ-9)	Dianhydro-aurasperone C, fonsecinone A, asperazine, rubrofusarin B, and (*R*)-3- hydroxybutanonitrile	*E. coli, B. subtilis, B. cereus*, and *S. aureus* (MICs = 25–50 μM)	(Xiao et al. 2014)
*A. fumigatiaffinis*	Neosartorin	*S. aureus, B. subtilis*, and most streptococci (MICs = 4–8 μg ml^−1^)	(Ola et al. 2014)
*Aspergillus* sp.	Ergosterol, cerevisterol, 5-hydroxymethylfuran-3-carboxylic acid, allantoin, trypacidin, and monomethylsulochrin	Allantoin showed the most activity, with MIC of 1 μg ml^−1^ against *S. aureus, P. aeruginosa, S. typhimurium*, and 2 μg ml^−1^ against *B. subtilis, Staphylococcus faecalis*, and *E. coli*	(Zhang et al. 2014).
*A. terreus* (SM-EF 3)	Ethyl acetate extract of mycelia	*S. typhi*, *S. aureus, V. cholera, E. coli, K. pneumoniae, S. paratyphi*, and *Klebsiella oxytoca* (IZDs = 11.6, 10.3, 10, 9.3, 9, 8.6, and 5.6 mm, respectively)	(Kalyanasundaram et al. 2015)
*A. flavus* (SM-EF 2)	Ethyl acetate extract of mycelia	*S. paratyphi, P. mirabilis*, and *S. aureus* (IZDs = 7, 5.3, and 5.6 mm, respectively)	(Kalyanasundaram et al. 2015)
*Aspergillus* sp. (IFB-YXS)	Xanthoascin	*Clavibacter michiganense* subsp. *sepedonicus* (MIC = 0.31 μg ml^−1^)	(Zhang et al. 2015)
*A. niger*	Ethyl acetate extract of mycelia	*S. aureus, B. subtilis*, and *E. coli* (IZDs = 9, 15, and 7 mm, respectively)	(Ratnaweera et al. 2015)
*A. fumigatus* (AF3-093A)	Flavipin, chaetoglobosin A, and chaetoglobosin B	*S. aureus*, MRSA, and *M. tuberculosis* (H37Ra)	(Flewelling et al. 2015)
*A. terreus*	(*22E,24R*)-stigmasta-5,7,22-trien-3-β-ol (38)	MRSA (IC_50_ = 0.96 μg ml^−1^)	(Ibrahim et al. 2015b)
*A. persii* (EML-HPB1-11)	Penicillic acid	Twelve phytopathogenic bacteria (MICs = 12.3–111.1 μg ml^−1^)	(Nguyen et al. 2016)
*A. awamori* (WAIR120; LC032125)	Emodin	*E. faecalis* (AHR7) (MIC = 125 μg ml^−1^)	(Ismaiel et al. 2016)
*A. tubingensis*	Ethyl acetate extract of the culture filtrate	*B. subtilis, S. aureus, P. aeruginosa, P. vulgaris, S. flexneri*, and *K. pneumonia* (IZDs = 20.3, 16.5, 18, 15.7, 13, and 15.6 mm, respectively)	(Padhi et al. 2017)
*A. tamarii* (SRRC 108818S)	Ethyl acetate extract of mycelia	*S. typhi* (ATCC33458), *S. aureus* (ATCC6538), *B. subtilis* (ATCC6633), *E. coli* (ATCC25922) (IZDs = 15.5, 14.5, 14, and 23 mm, respectively)	(Ogbole et al. 2017)
*A. fumigatus*	Acetonitrile extract of the culture filtrate	*S. typhimurium, Listeria monocytogenes*, and *P. aeruginosa* (IZDs = 8, 19, and 25 mm, respectively)	(Hassan and Bakhiet 2017)
*A. fumigatus* and *A. niger*	Ethyl acetate extract of mycelia	*Proteus mirabilis, S. aureus, K. pneumoniae, P. aeruginosa, B. subtilis*, and *P. fluorescense* (IZDs of *A. fumigatus *= 9, 9, 6, 13, 8, and 6.5 mm, respectively, while IZDs of *A. niger *= 2–6 mm)	(Akinyemi 2017)
*A. flavus, A. fumigatus*, and *A. niger*	Ethyl acetate extract of the culture filtrate	*S. pneumoniae, S. aureus, E. coli*, and *P. aeruginosa* (highest activity was of *A. fumigatus*, with MICs of 250 μg ml^−1^)	(Yahaya et al. 2017)
*A. clavatonanicus* (MJ31)	Ethyl acetate extracts of the mycelia and culture filtrate	*B. subtilis, M. luteus*, and *S. aureus* (MICs = 0.078, 0.156, and 0.312 mg ml^−1^, respectively)	(Mishra et al. 2017)
*A. niger* (MTCC-961)	Ethyl acetate extract of the culture filtrate	Gram-positive and Gram-negative bacteria (IZDs = 11–18 mm)	(Kalyani and Hemalatha 2017)
*A. oryzae* (DBM4336)	Ethyl acetate and ethanolic extracts of mycelia	Gram-positive and Gram-negative bacteria (IZDs = 1–10 mm by ethanolic)	(Synytsya et al. 2017)
*Aspergillus* spp.	Chloroform extract of the culture filtrate	*S. aureus* (ATCC 25923), *B. subtilis* (ATCC 6633), and *E. coli* (ATCC 25922) (IZDs = 7–31.67 mm)	(Amina et al. 2017)
*A. sclerotiorum* (PSU-RSPG178)	Penicillic acid	*S. aureus* and *E. coli* (MICs = 128 μg ml^−1^, for each)	(Phainuphong et al. 2017)
*A. niger*	Ethyl acetate extract of mycelia	*S. aureus* (MTCC96), *M. luteus* (MTCC106), *P. aeruginosa* (MTCC326), *E. faecalis* (MTCC439), and *P. mirabilis* (MTCC1429) (IZDs = 15–23 mm)	(Thorati and Mishra 2017)
*Aspergillus* sp.	Ethyl acetate extract of the culture filtrate	Gram-positive and Gram-negative bacteria (IZDs = 9.2–15.2 mm and MICs = 15.62–250 μg ml^−1^)	(Monggoot et al. 2018).
*A. nidulans* (MA-143)	Isoversicolorin C, isosecosterigmatocystin, glulisine, and versicolorin	Highest activities were for isoversicolorin C against *Vibrio alginolyticus* and *E. ictaluri* (MICs = 1 and 4 μg ml^−1^, respectively) and for Isosecosterigmatocystin against *E. coli, M. luteus, V. alginolyticus, V. parahaemolyticus*, and *E. ictaluri* (MICs = 1–8 μg ml^−1^)	(Yang et al. 2018)
*A. versicolor*	Bisabolane sesquiterpenoid derivatives	*E. carotovora* subsp. *carotovora* (MICs = 15.2 to 85.2 μg ml^−1^	(Guo et al. 2018)
*A. fumigatus* (CBA2743)	Unidentified fractions of the fermentation broth	F4 fraction fraction had positive antimycobacterial activity (MIC = 256 μg ml^−1^)	(Silva et al. 2018)

Wilkins and Harris (1942) examined 100 species of fungi for the production of bacteriostatic or bactericidal substances. About 40% of the *Aspergillus* strains yielded antibiotics. *A. fumigatus* mut. *helvola* yielded particularly active filtrates, apparently superior to the bacteriostatic substances produced by *Penicillium*. The tested bacteria were *E. coli, Staphylococcus aureus*, and *Pseudomonas aeruginosa*. A year later, Chain et al. (1943) subsequently isolated a crystalline antibiotic against Gram-positive bacteria from *Aspergillus* filtrates, which was named helvolic acid (1), and the name being derived from the variety of *A. fumigatus* yielding the best product.

A penicillin-like substance was isolated from *A. giganteus* (Wehm) by Philpot (1943), which she named gigantic acid. Later, Florey et al. (1944) isolated the antibiotic claviformin (patulin) (2) from a solution produced by *A. giganteus* (Wehm). For many years, claviformin was originally used as an antibiotic against Gram-positive and Gram-negative bacteria, but due to its toxicity to humans and animals, it was reclassified as a mycotoxin during the 1960s (Bennett and Klich 2003).

In 1944, Soltys (1944) extracted a product from *A. fumigatus* called aspergillin (3). This product is inactive against staphylococci but inhibits the growth of *Mycobacterium tuberculosis* even when tested in high dilutions. He concluded that this antibacterial is nontoxic for experimental animals and is sufficiently stable to withstand boiling for one hour.

**Figure:**
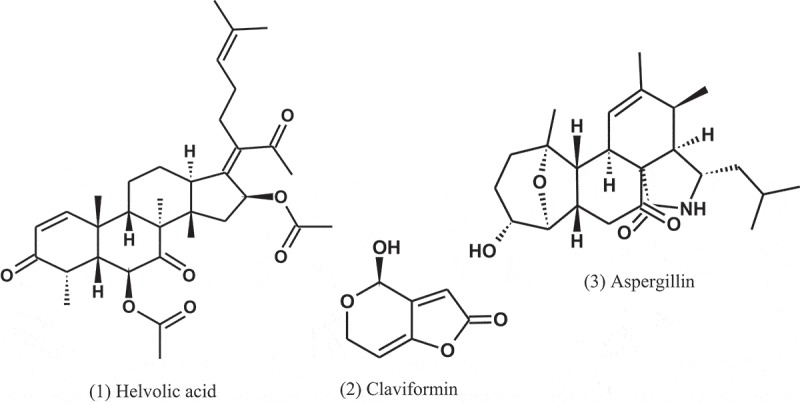

Fumagillin (4) is a compound first isolated in 1949 as an antiphage agent called as antibiotic H-3 (Hanson and Eble 1949). This compound was isolated from *A. fumigatus* (H-3), and subsequently it was found to have antibacterial property, and was named fumagillin (Eble and Hanson 1951). After its discovery, fumagillin was used to treat human intestinal amebiasis (Killough et al. 1952) and microsporidiosis caused by *Enterocytozoon bieneusi* (Conteas et al. 2000).

**Figure:**
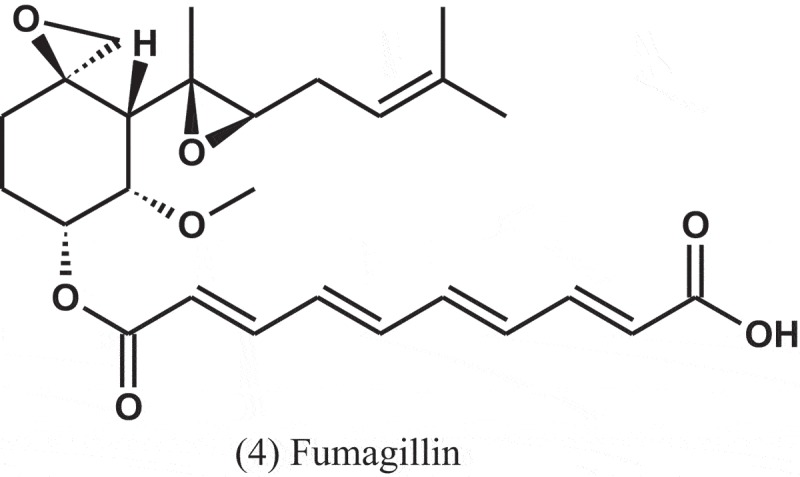

*A. caespitosus* (NRRL 5769), growing in broth containing small amounts of sitosterol, produced three metabolites, namely, 5,6-dihydro-5(S)-acetoxy-6(S)-1,2-trans-epoxypropyl)-2H-pyran-2-one (5), 5,6-dihydro-5(S)-acetoxy-6(S)-(1,2-trans propenyl)-2H-pyran-2-one (6), and 5,6-dihydro-5(R)-acetoxy-6(S)-(1,2-trans-epoxy-propyl)-H-pyran-2-one (7). These metabolites showed antibacterial activity against *Salmonella paratyphi* A, with MIC values of 50, 50, and 100 μg ml^−1^, respectively (Mizuba et al. 1975).

**Figure:**
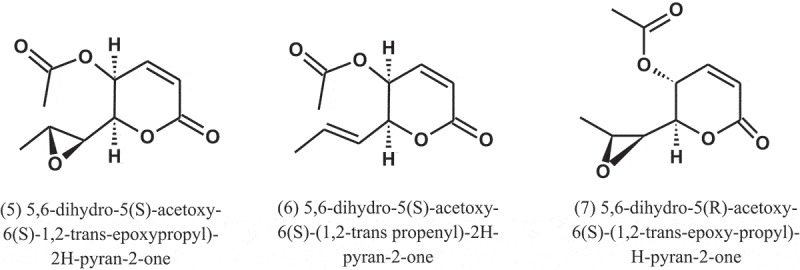

Dihydrogeodin (8) was isolated from the fungus *A. terreus* var. *aureus* and showed antibacterial activity against *Bacillus subtilis* (IFO-3513), with MIC value of 6.25 μg ml^−1^ (Inamori et al. 1983).

A compound, named as CJ-17,665 (9), was isolated from the fermentation broth of *A. ochraceus* (CL41582), which isolated from a soil sample collected in Venezuela. This compound showed inhibitory effect against multi-drug resistant *S. aureus* (MDRSA), *S. pyogenes*, and *E. faecalis*, with MIC values of 12.5, 12.5, and 25 μg ml^−1^, respectively (Sugie et al. 2001). The chemical structure of this compound contains a diketopiperazine and an indole N-oxide moiety.

**Figure:**
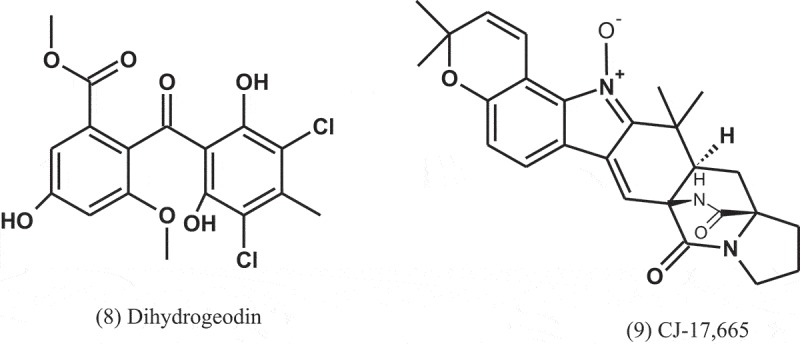

The chloroform extracts of *A. fumigatus*, isolated from a soil sample collected in Pantanal, Brazil, showed antibacterial activity against *S. aureus* and *M. luteus*, with IZDs of 20.7 and 39.3 mm, respectively. The antibacterial activity detected for the chloroform extract of the culture broth incubated without the pool of bacteria was lower than that detected for the extract with the pool. Benzathin penicillin G was used as positive control, with IZDs of 33 and >40 mm for *S. aureus* and *M. luteus*, respectively. The active compounds were isolated from the broth of the culture grown in the presence of pooled bacteria and identified as 3,4-dimethoxyphenol (10) and 1,3,5-trimethoxybenzene (11) (Furtado et al. 2002).

**Figure:**
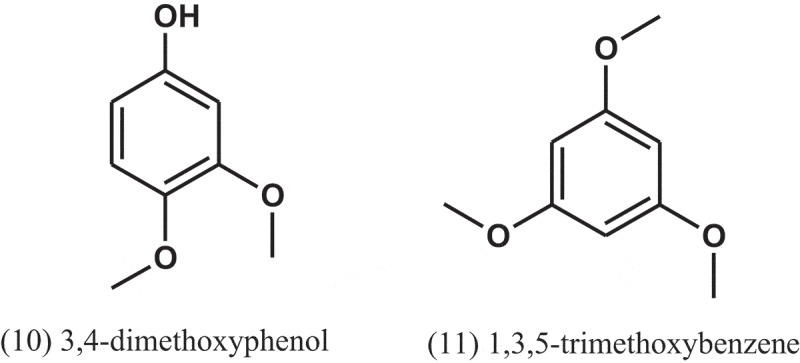

Fractionation of the extract of *A. niger* (IFB-E003), an endophyte in *Cyndon dactylon* (Poaceae), led to isolation of naphtho-γ-pyrones: rubrofusarin B (12), fonsecinone A (13), asperpyrone B (14), and aurasperone A (15). The four compounds exhibited growth inhibitions against *B. subtilis, E. coli*, and *P. fluorescence*, with MICs ranging from 1.9 to 31.2 μg ml^−1^. Penicillin and amikacin sulphate were used as positive controls (MICs = 0.45–3.9 μg ml^−1^) (Song et al. 2004).

**Figure:**
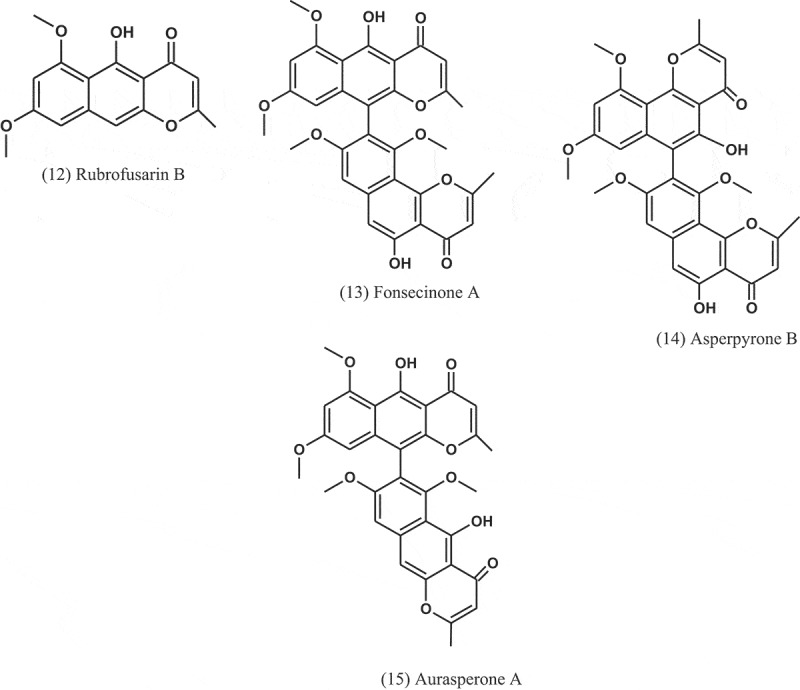

*Aspergillus* sp. (CY 725) was isolated from leaves of *C. dactylon* (Poaceae). Through a bioassay-guided fractionation of the ethyl acetate extract of this fungus, four secondary metabolites were isolated. These metabolites were identified as helvolic acid (1), monomethylsulochrin (16), ergosterol (17), and 3β-hydroxy-5α,8α-epidioxy-ergosta-6,22-diene (18). These compounds showed antibacterial activity against *Helicobacter pylori*, but helvolic acid was the most active, with MIC value of 8 μg ml^−1^ (Li et al. 2005).

**Figure:**
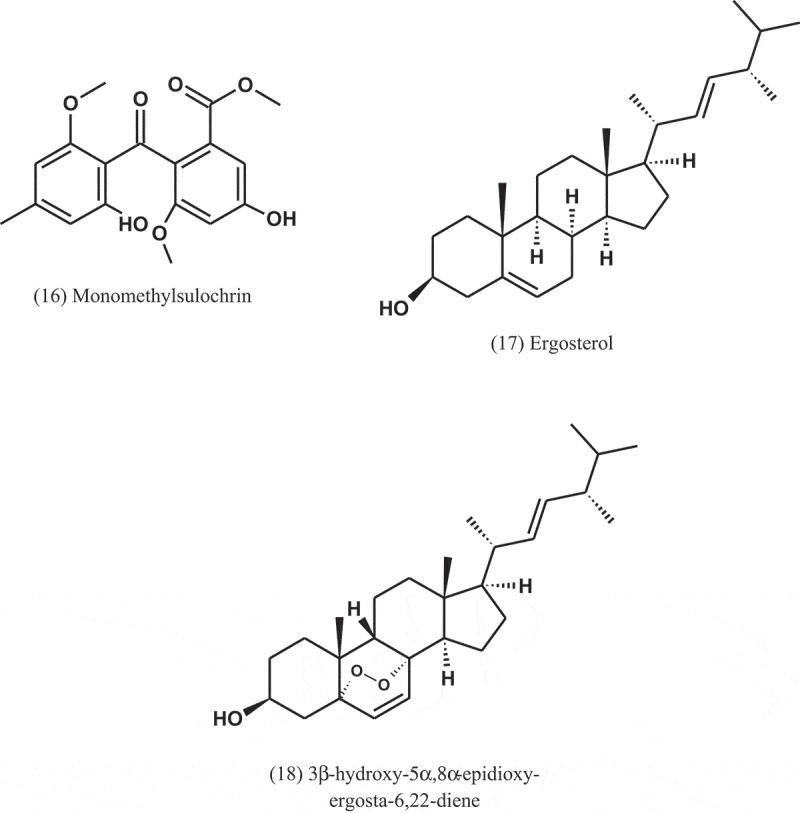

The fungus *A. terreus* var. *terreus* isolated from an Ecuador soil sample was cultured in liquid and solid media and yielded metabolites, including terreic acid (19) and butyrolactone I (20). These metabolites were screened for their antibacterial activity using agar diffusion assay, at a concentration of 100 μg disc^−1^, and serial dilution technique using 96-well microtitre plates. Compounds 19 and 20 were active towards the phytopathogenic bacterium *Erwinia carotovora*, with IC_50_ of 5.1 and 12.5 μg ml^−1^, respectively, compared to streptomycin (IC_50_ = 1.9 μg ml^−1^). Compound 19 was also active against *B. subtilis* and *Micrococcus luteus*, with IZDs of 35 and 8 mm, respectively, compared to chloramphenicol (IZDs = 26 and 36 mm, respectively). Compound 20 also inhibited the growth of *Bacillus brevis, B. subtilis*, and *Enterobacter dissolvens*, with IZDs of 30, 21, and 17 mm, respectively, compared to chloramphenicol (IZDs = 25, 26, and 21 mm, respectively) (Cazar et al. 2005).

**Figure:**
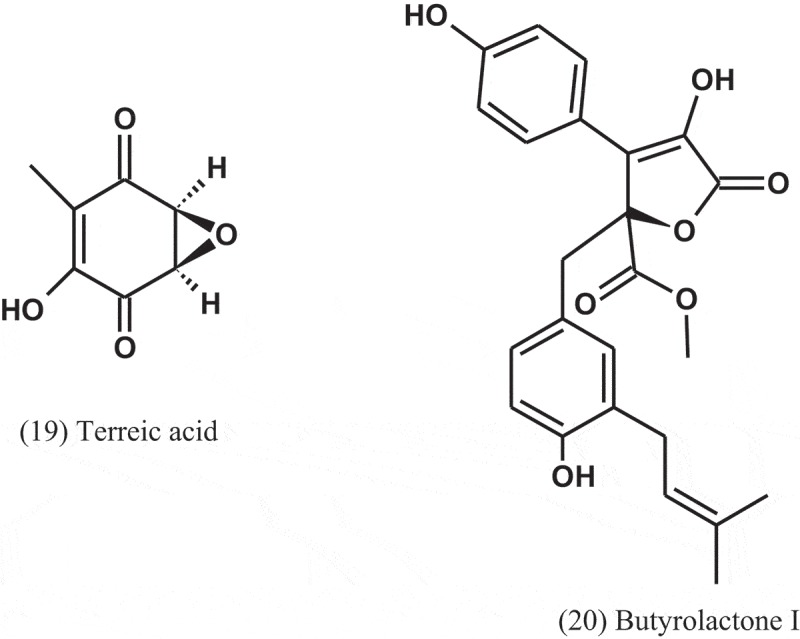

It is well known that kojic acid (21), produced by various fungi including *Aspergillus* spp., is reported to possess antibacterial activity (Wilkins and Harris 1942; Marwaha et al. 1994). In a study conducted by El-Aasar (2006), *A. flavus, A. glaucus, A. oryzae, A. parasiticus*, and *A. tamirii*, isolated from a soil, were cultured for kojic acid production. The antibacterial activity of this compound was tested against six bacterial pathogens including Gram-positive and Gram-negative bacteria. The results revealed that kojic acid has activity against all the tested bacteria, with MIC values of 176–285 μg ml^−1^. Ceftazidime (MIC = 14–34 μg ml^−1^) and nitrofurantoin (MIC = 65–105 μg ml^−1^) were used as positive controls.

Six alkylitaconic acids, designated tensyuic acids A to F, were isolated from the culture broth of *A. niger* (FKI-2342) isolated from a soil collected at Ooura Tensyudou, Nagasaki, Japan. Only tesyuic acid C (22) showed antibacterial activity against *B. subtilis*, with IZD of 10 mm, at a concentration of 50 μg disc^−1^ (Hasegawa et al. 2007).

Emodin (23) was purified and elucidated from the ethyl acetate extract of fermentation broth of the fungus *A. awamori* (F12), which isolated from rhizospheric soil of *Rhizophora stylosa* (Rhizophoraceae). Antibacterial evaluation of emodin showed activity against *S. aureus* and *B. subtilis*, with MIC values of 16 and 32 μg ml^−1^, respectively (Chang et al. 2010).

**Figure:**
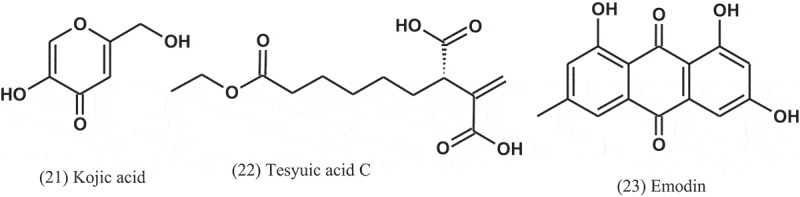

The alkaloids known as fumigaclavine C (24) and pseurotin A (25) were isolated from *Aspergillus* sp. (EJC08) of the plant *Bauhinia guianensis* (Fabaceae). Compound 24 showed antibacterial activity against *B. subtilis, E. coli, P. aeruginosa*, and *S. aureus*, with MIC values of 7.8, 62.5, 31.3, and 15.6 μg ml^−1^, respectively, while Compound 25 was active against *B. subtilis, E. coli, P. aeruginosa*, and *S. aureus*, with MIC values of 15.6, 31.3, 31.3, and 15.6 μg ml^−1^, respectively (Pinheiro et al. 2013).

**Figure:**
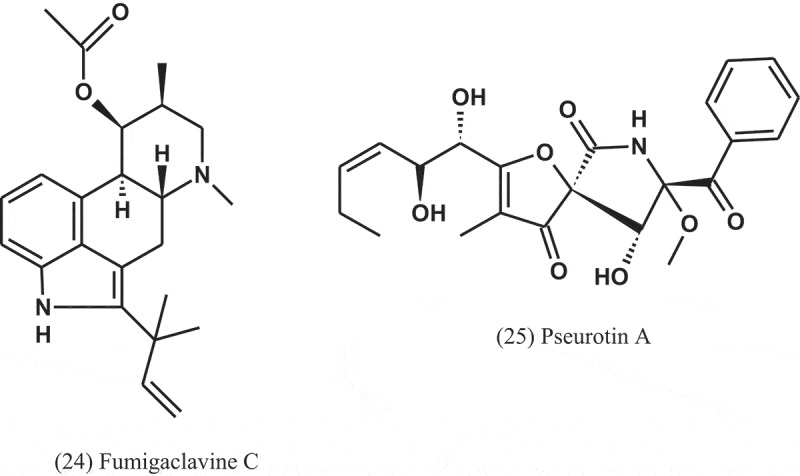

Dianhydro-aurasperone C (26), fonsecinone A (11), asperazine (27), rubrofusarin B (12), and (*R*)-3- hydroxybutanonitrile (28) were isolated from the fungal endophyte *Aspergillus* sp. (KJ-9) obtained from the plant *Melia azedarach* (Meliaceae) and tested against four bacteria: *E. coli, B. subtilis, B. cereus*, and *S. aureus*. These compounds displayed weak to moderate antibacterial activities against some bacteria (MICs = 25–50 μM) and exhibited better activity against Gram-positive bacteria than Gram-negative bacteria. Fonsecinone A inhibited the growth of *S. aureus* and *B. subtilis*, with equal MIC values of 25 μM, but weaker than that of streptomycin sulphate (MIC = 12.5 μM) (Xiao et al. 2014)

**Figure:**
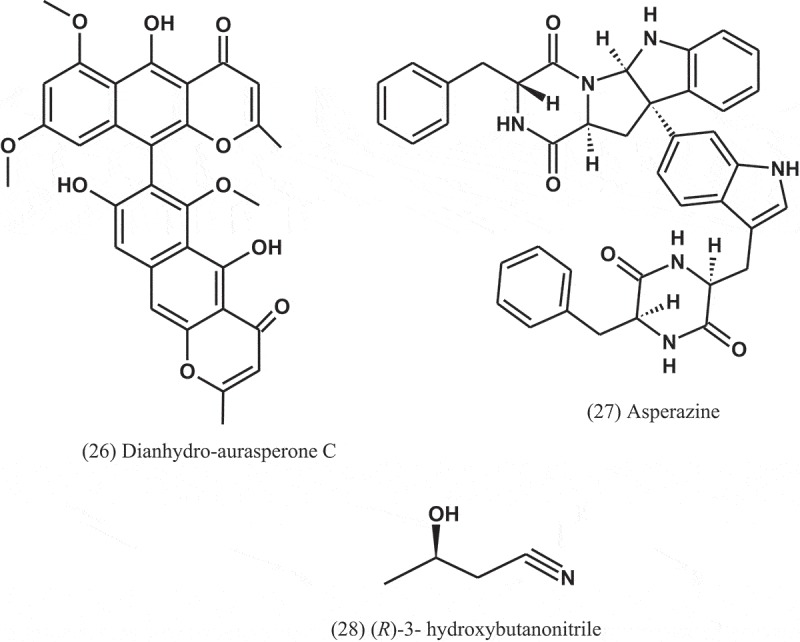

.

Neosartorin (29) was isolated from the endophytic fungus *A. fumigatiaffinis*, which isolated from the plant *Tribulus terrestris* (Zygophyllaceae) collected in Uzbekistan. Compound 29 inhibited the growth of a broad spectrum of Gram-positive bacteria, while the tested Gram-negative species were not affected. MIC values from4 to 8 μg ml^−1^ were obtained for *S. aureus, B. subtilis*, and most streptococci and the growth of enterococci was inhibited at slightly higher concentrations (16–32 μg ml^−1^) (Ola et al. 2014).

**Figure:**
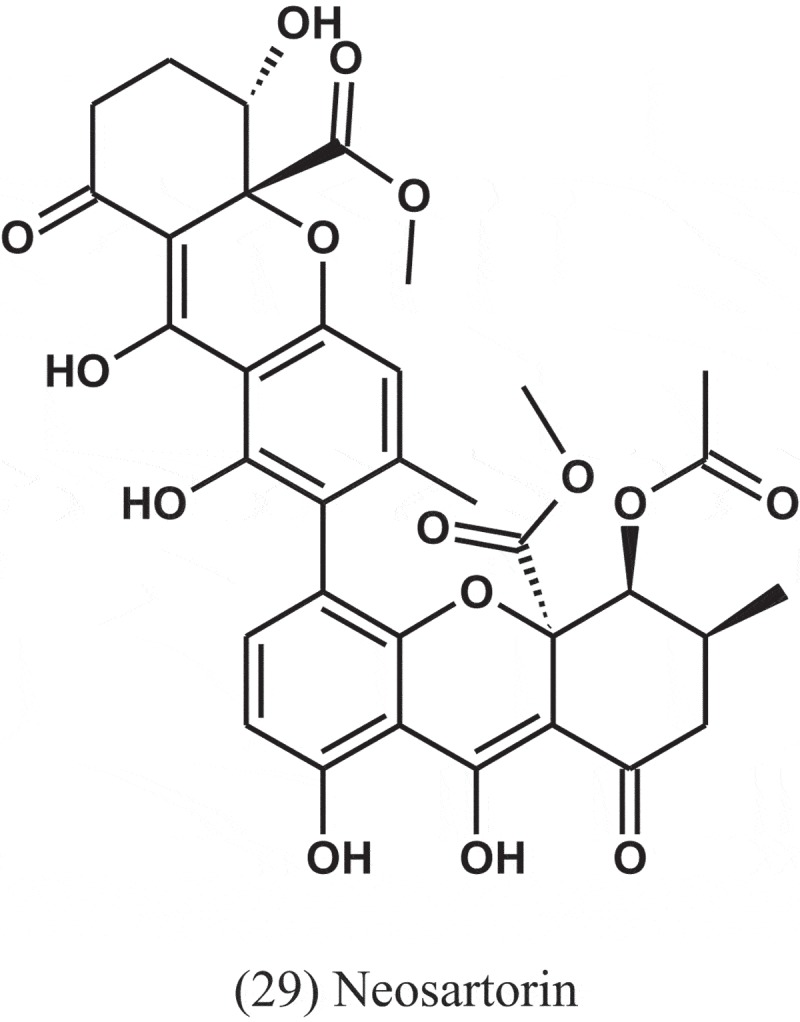

Seven compounds, namely, ergosterol (17), cerevisterol (30), 5-hydroxymethylfuran-3-carboxylic acid (31), allantoin (32), trypacidin (33), and monomethylsulochrin (16) were isolated from the endophytic fungal strain *Aspergillus* sp. (ER15), which obtained from the roots of *Eucommia ulmoides* (Eucommiaceae). These compounds were tested against a panel of Gram-positive and Gram-negative bacteria. Among these compounds, compound 32 was the most active, with MIC values of 1 μg ml^−1^ against *S. aureus, P. aeruginosa, S. typhimurium*, and 2 μg ml^−1^ against *B. subtilis, Staphylococcus faecalis*, and *E. coli* (Zhang et al. 2014).

**Figure:**
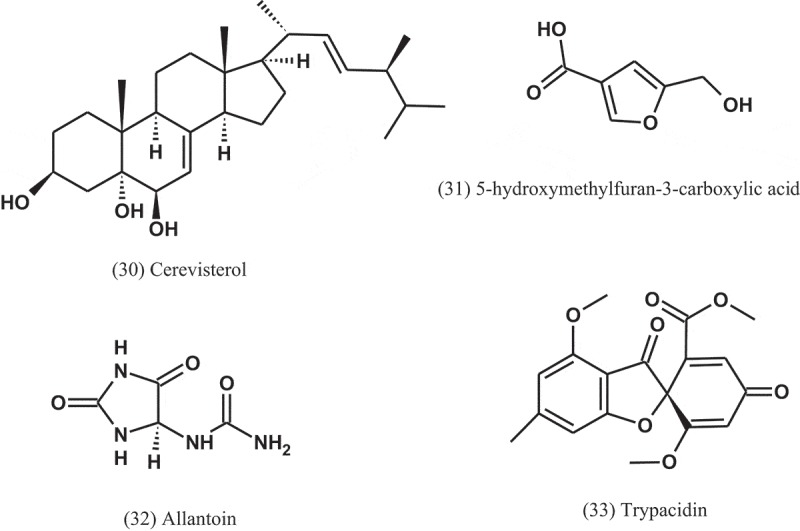

Zhang et al. (2015) also tested the antibacterial activity of phenolic compounds obtained from ethanol extract derived from the solid-substrate fermentation of *Aspergillus* sp. (IFB-YXS), an endophytic fungus residing in the leaves of *Ginkgo biloba* (Ginkgoaceae). Among the compounds, xanthoascin (34) significantly inhibited the growth of the phytopathogenic bacterium *Clavibacter michiganense* subsp. *sepedonicus*, with MIC value of 0.31 μg ml^−1^, which is more potent than the positive control streptomycin (MIC = 0.62 μg ml^−1^). The antibacterial mechanism of xanthoascin was attributed to change the cellular permeability of the phytopathogens, leading to the remarkable leakage of nucleic acids out of the cytomembrane.

**Figure:**
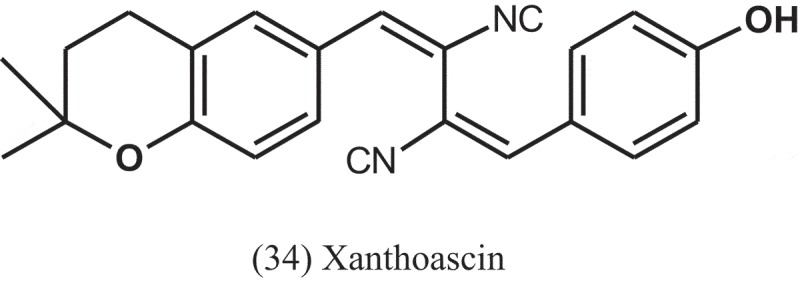

The crude extract of *A. fumigatus* (AF3-093A), an endophyte of the brown alga *Fucus vesiculosus* (Fucaceae), displayed significant antibacterial activity in initial bioactivity screens. Bioassay-guided fractionation of the extract led to the isolation of flavipin (35), chaetoglobosin A (36), and chaetoglobosin B (37). These compounds inhibited the growth of *S. aureus*, MRSA, and *M. tuberculosis* (H37Ra) (Flewelling et al. 2015).

**Figure:**
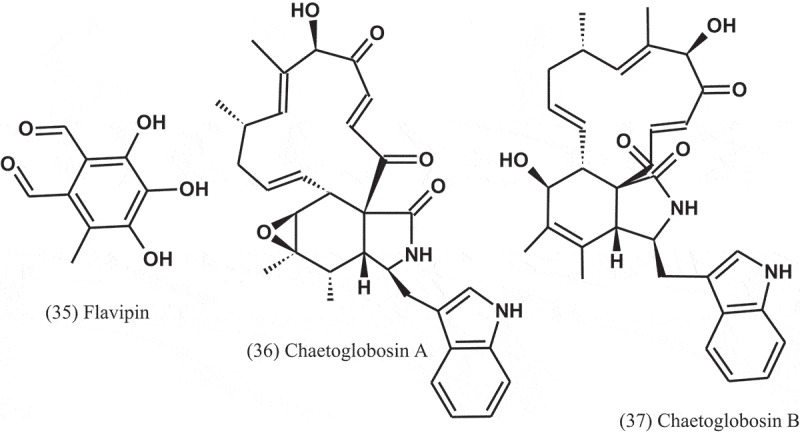

Eight compounds were isolated from the endophytic fungus *A. terreus* isolated from the roots of *Carthamus lanatus* (Asteraceae) (Ibrahim et al. 2015b). The compound (*22E,24R*)-stigmasta-5,7,22-trien-3-β-ol (38), a stigmasterol derivative, displayed a potent activity against MRSA with IC_50_ value of 0.96 μg ml^−1^, compared to ciprofloxacin (IC_50_ = 0.07 μg ml^−1^).

The seed-borne fungus *A. persii* (EML-HPB1-11), isolated from the seeds of barley (Poaceae), produced penicillic acid (3-methoxy-5-methyl-4-oxo-2,5-hexadienoic acid) (39), which tested against 12 phytopathogenic bacteria. All of the bacterial pathogens tested were highly inhibited by this compound, with MIC values of 12.3–111.1 μg ml^−1^ (Nguyen et al. 2016). Although penicillic acid has antibacterial activity, it is toxic to human cells and halogenated furanone has carcinogenic toxicity (Bjarnsholt and Givskov 2007).

**Figure:**
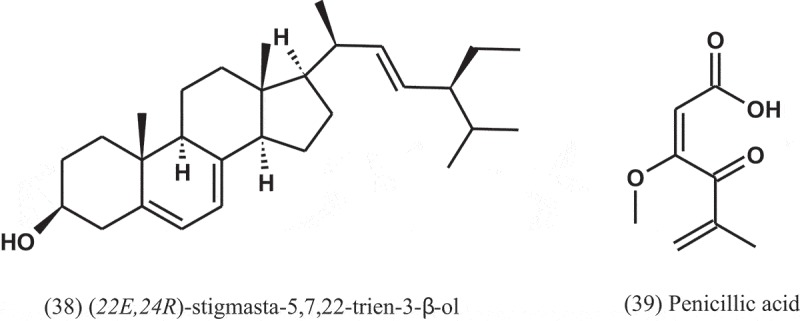

In a study conducted by Ismaiel et al. (2016), emodin (23) was isolated from *A. awamori* (WAIR120; LC032125), which isolated from stored wheat grain sample obtained locally from a retail supermarket (Zagaig, Egypt). This compound showed antibacterial activity against *E. faecalis* (AHR7), with MIC of 125 μg ml^−1^. Emodin was found to induce morphogenic effects including swelling and elongation of bacterial cell as shown by light microscopy. In addition, cellular effects were also resulted, in which emodin caused considerable changes in the nature of cell membrane and submicroscopic structure of bacterial cell as shown by transmission electron microscopy.

Ethyl acetate extracts of the fungi *A. flavus, A. fumigatus*, and *A. niger*, isolated from the soil of Bayero University Kano, were investigated for their antibacterial activities by disc diffusion assay. The extracts showed inhibitory activity against *S. pneumoniae, S. aureus, E. coli*, and *P. aeruginosa*. Highest activity was observed from the metabolites of *A. fumigatus* on all the test bacteria, with MIC values of 250 μg ml^−1^. The chromatogram study reveals many bioactive compounds, such as oleic acid (40) and n-hexadecanoic acid (41) known to have antibacterial activity against range of bacteria (Yahaya et al. 2017).

**Figure:**
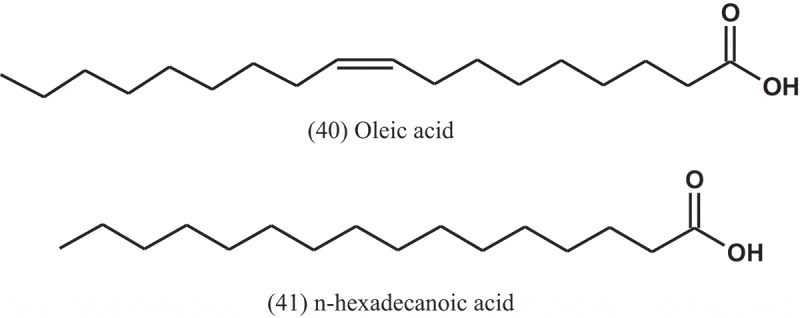

Phainuphong et al. (2017) found that penicillic acid (39), isolated from the fungus *A. sclerotiorum* (PSU-RSPG178), obtained from a soil sample from Suratthani province, Thailand, has antibacterial activity against *S. aureus* and *E. coli*, with equal MIC values of 128 μg ml^−1^.

Many strains of *Aspergillus* sp. were isolated and cultured from the flowers of the ethnomedicinal plant *Mitrephora wangii* (Annonaceae). The extract of the strain *Aspergillus* sp. (MFLUCC16-0845) showed broad activity against many bacterial pathogens, including *S. aureus* (ATCC 25923), *S. epidermidis* (ATCC 12228), *S. agalactiae* (ATCC13813), *Bacillus subtilis* (ATCC 19659), *B. cereus* (ATCC 11778), *E. coli* (ATCC 25922), *S. typhi* (ATCC 14028), *K. pneumoniae* (ATCC 700603), *P. aeruginosa* (ATCC 27853), and *S. flexneri* (ATCC 12022), with IZDs and MICs values of 8.3–13.4 mm and 15.62–500 μg ml^−1^, respectively. Penicillin was used as a positive control and exhibited IZDs and MICs of 9.2–15.2 mm and 15.62–250 μg ml^−1^, respectively. The results of gas chromatography–mass spectrometry indicated to the presence of many fungal metabolites, but β-thujaplicin (42) was at high yield (Monggoot et al. 2018).

Four compounds, namely isoversicolorin C (43), isosecosterigmatocystin (44), glulisine (45), and versicolorin C (46) were isolated from the mangrove-derived endophytic fungus *A. nidulans* (MA-143), obtained from leaves of the plant *R. stylosa*. Using the microplate assay, these compounds were tested for antibacterial activities against three human pathogens (*E. coli, M. luteus*, and *Vibrio vulnificus*) and four aquatic bacteria (*Edwardsiella ictaluri, Vibrio alginolyticus, V. anguillarum*, and *V. parahaemolyticus*). Compound 43 showed potent antibacterial activity against *V. alginolyticus* and *E. ictaluri*, with MIC values of 1 and 4 μg ml^−1^, respectively, while compound 44 exhibited activity against *E. coli, M. luteus, V. alginolyticus, V. parahaemolyticus*, and *E. ictaluri*, with MIC values ranging from 1 to 8 μg ml^−1^. Chloramphenicol was used as a positive control and exhibited MIC values ranging from 0.5 to 8 μg ml^−1^ (Yang et al. 2018).

**Figure:**
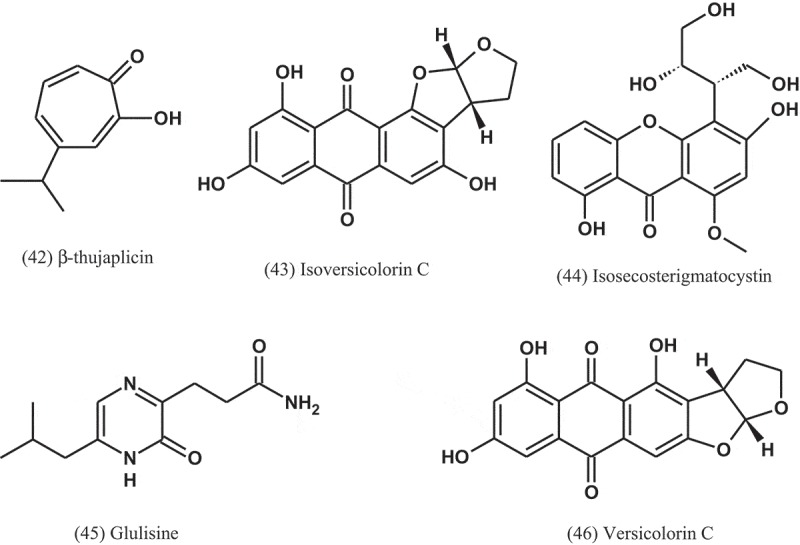

Twelve bisabolane sesquiterpenoid derivatives were isolated from the endophytic fungus *A. versicolor*, obtained from the leaves of *Elaeocarpus decipiens* (Elaeocarpaceae). The activity of these compounds were tested against the enterobacterial plant pathogen *E. carotovora* subsp. *carotovora*, and the results showed that all compounds displayed antibacterial activities, with MIC values ranging from 15.2 to 85.2 μg ml^−1^ (Guo et al. 2018).

Under specific culture conditions, the endophytic *A. fumigatus* (CBA2743), obtained from the plant *Copaifera multijuga* (Fabaceae), released metabolites into the fermentation broth. The fractions of the fermentation broth were obtained by different chromatographic techniques and tested against *Mycobacterium smegmatis* and *M. tuberculosis*. The results showed that F4 fraction had positive antimycobacterial activity, with MIC value of 256 μg ml^−1^ (Silva et al. 2018).

### 3.2. Antibacterial properties of crude extracts

In this section, we discuss the crude extracts having antibacterial activities from terrestrial *Aspergillus* spp. These extracts with their susceptible bacteria are listed in Table 1. In preparing enzyme concentrates from certain moulds, antibacterial activity was noted in extract from a number of species characterized by high proteolytic capacity. Thus, two strains of *A. clavatus* were able to synthesise and release bactericidal products, into the liquid media, which possessed antibacterial activities against *S. aureus* and other bacteria (Wiesner 1942). In a study conducted by Foster and Karow (1945), antibacterial substances present in culture filtrates of *A. niger* (YWA), *A. nidulans, A. oryzae* (TP), and *A. flavipes* are either identical with, or closely related to, authentic penicillin produced by *Penicillium notatum*. This was shown by solubility properties, thermostability, pH stability, antibacterial spectra against 18 different bacteria, destruction by the enzyme penicillinase, and (in one instance) by efficacy in protecting animals against a lethal bacterial infection.

Wilkins and Harris (1945) observed that *Aspergillus* spp. produced bacteriostatic substances against *Bacterium coli, S. aureus*, and *Pseudomonas pyocyanea*.

Brian and Hemming (1947) reported that the culture filtrates of different species of *Aspergillus* displayed antibacterial activity against *S. aureus* and *Salmonella typhi*.

Irobi et al. (2000) worked on *A. quadrilineatus* isolated from moldy Acha, a common cereal grown among the plateau state of Nigeria. They found that the petroleum ether defatted crude extract of this fungus has remarkable antibacterial activity against *S. aureus* and *B. Subtilis*, with IZDs of 18 and 20 mm, respectively at pH 6.4.

Antibacterial activity of crude and partially purified ethyl acetate extracts, derived from two species of endophytic fungus *Aspergillus*, was tested. These fungi were isolated from mangrove fern *Acrostichum aureum* and mangrove angiosperm associate, *Acanthus ilicifolius* of Nethravathi Mangrove, on the southwest coast of India. The results showed different activities against a panel of bacteria including Gram-negative and Gram-positive bacteria, with IZDs ranging from 0 to 15.2 mm (Maria et al. 2005).

The antibacterial activity of the mycelia extracts and crude culture broth of the endophytic fungus *Aspergillus* sp., isolated from the leaf samples of *Justicia adathoda* (Acanthaceae), was evaluated by agar well diffusion method against some bacterial strains. Crude mycelial extract inhibited all the strains significantly, with a mean strongest IZD of 12 mm against *E. coli*. Crude culture broth inhibited the strains of *P. aeruginosa* and *Klebsiella pneumoniae* alone and had a mean stronger IZD of 13 and 15 mm, respectively, than crude mycelial extract (Prabavathy and Nachiyar 2012).

The crude ethyl acetate extract of *A. fumigatus*, isolated from the plant *Garcinia* spp. (Clusiaceae), was screened for antibacterial activity. It showed antibacterial activities against *B. subtilis, E. coli, K. pneumoniae, Shigella flexneri*, and *S. aureus*, with IZDs of 16, 18, 20, 11, and 16 mm, respectively (Ruma et al. 2013).

Al-Shaibani et al. (2013) studied the inhibitory effect of the culture filtrate of *A. niger*, obtained from the inflamed eyes of patients against *P. aeruginosa, S. aureus, S. epidermidis*, and *Bacillus* sp., which isolated from patients of microbial keratitis, who attended to Ibn Al-Haetham Eye Teaching Hospital, Baghdad, Iraq. The results revealed that *A. niger* possessed inhibitory effect against *P. aeruginosa, S. aureus, S. epidermidis*, and *Bacillus* sp., with IZDs of 15, 25, 30, and 32 mm, respectively.

*Aspergillus* sp. was isolated from the roots and leaves of *Triticum durum* (Poaceae) collected from the Bordj Bou Arreridj region (Algeria). Antibacterial activity of the crude ethyl acetate extract of this fungus was evaluated using agar diffusion assay against 12 pathogenic bacteria. Except *Enterobacter agglomerans, P. aeruginosa*, and *Citrobacter freundii*, the results revealed inhibition activity against the tested bacteria, with IZDs of 11.3–21.7 mm, and the largest zone was 21.7 mm against *E. coli* (Sadrati et al. 2013).

Many endophytic fungi were isolated from the leaves and stems of the plants *Suaeda maritima* and *S. monoica* (Amaranthaceae). Among these fungi, *A. terreus* (SM-EF 3) and *A. flavus* (SM-EF 2) were isolated. The fungal culture of these fungi were extracted with ethyl acetate and used as crude extract for checking their antibacterial activities by well diffusion method. The extract of *A. terreus* showed antibacterial activity, with IZDs of 11.6, 10.3, 10, 9.3, 9, 8.6, and 5.6 mm against *S. typhi, S. aureus, V. cholera, E. coli, K. pneumoniae, S. paratyphi*, and *Klebsiella oxytoca*, respectively. The extract of *A. flavus* showed antibacterial activity against *S. paratyphi, P. mirabilis*, and *S. aureus* with IZDs of 7, 5.3, and 5.6 mm, respectively (Kalyanasundaram et al. 2015).

The crude extract of the endophytic fungus *A. niger*, isolated from the leaves of *Opuntia dillenii* (Cactaceae), was investigated for its antibacterial potential against five bacterial species. The fungal extract showed activity against *S. aureus, B. subtilis*, and *E. coli*, with IZDs of 9, 15, and 7 mm, respectively (Ratnaweera et al. 2015).

The crude extract obtained from endolichenic fungus *A. tubingensis*, isolated from surface-sterilised lichen thallus of *Parmelia caperata* (Parmeliaceae), was assayed against six clinically significant bacterial pathogens. The extract displayed considerable antibacterial activity against *B. subtilis, S. aureus, P. aeruginosa, P. vulgaris, S. flexneri*, and *K. pneumonia*, with IZDs of 20.3, 16.5, 18.0, 15.7, 13.0, and 15.6 mm, respectively (Padhi et al. 2017).

The endophytic fungus *A. tamarii* (SRRC 108818S), isolated from the mushroom *Lycoperdon umbrinum* (Agaricaceae), was investigated for its antibacterial activity. At a concentration of 200 mg ml^−1^, ethyl acetate extract of this fungus showed activity against *S. typhi* (ATCC33458), *S. aureus* (ATCC6538), *B. subtilis* (ATCC6633), and *E. coli* (ATCC25922), with IZDs of 15.5, 14.5, 14, and 23 mm, respectively. Gentamicin was used as a positive control with IZDs of 16–18 mm (Ogbole et al. 2017).

The ability of *A. fumigatus*, isolated from Sudanese indigenous soil, to produce antibacterial compounds was tested by Hassan and Bakhiet (2017). The crude extract (5%) of the fungus exerted activity against *Salmonella typhimurium, Listeria monocytogenes*, and *P. aeruginosa*, with IZDs of 8, 19, and 25 mm, respectively.

Using agar well diffusion method, the mycelial extracts of the fungal endophytes, *A. fumigatus* and *A. niger*, isolated from different medicinal plants, were investigated for antibacterial activity against some bacterial species. *A. fumigatus* showed inhibition, with IZDs of 9, 9, 6, 13, 8, and 6.5 mm against *Proteus mirabilis, S. aureus, K. pneumoniae, P. aeruginosa, B. subtilis*, and *P. fluorescense*, respectively, while *A. niger* showed lowest inhibition, with IZDs ranging from 2 to 6 mm. Tetracycline was used as a positive control, with IZDs ranging from 13 to 21 mm (Akinyemi 2017).

Mishra et al. (2017) observed that ethyl acetate extract of the endophytic fungus *A. clavatonanicus* (MJ31), isolated from roots of *Mirabilis jalapa* (Nyctaginaceae), displayed antibacterial potential against *B. subtilis, M. luteus*, and *S. aureus*, with MICs of 0.078, 0.156, and 0.312 mg ml^−1^, respectively.

At different concentrations, the fungal extract of *A. niger* (MTCC-961), obtained from Microbial Type Culture Collection, Chandigarh, India, was tested for its antibacterial activity against eight bacterial species by agar well diffusion method. The results displayed antibacterial activity, with IZDs ranging from 11 to 18 mm, at a concentration of 100 μg ml^−1^. It also observed that the activity was concentration dependent. Gentamicin was used as a positive control and exhibited IZDs ranging from 15 to 19 mm (Kalyani and Hemalatha 2017).

*A. oryzae* (DBM4336), obtained from the Department of Biochemistry and Microbiology (DBM), University of Chemistry and Technology (Prague, Czech Republic), was screened for its antibacterial activity. At different solvent extractions, the antibacterial properties of fungal extracts were screened against seven bacterial species by disc diffusion method. From the results of the study, it was observed that different solvents used for preparation of the mycelial extracts gave different degrees in inhibitory activity. For this, the ethanol extract was found to be active against all seven testing bacteria used, with IZDs ranging from 1 to 10 mm, while aqueous extract was found to be not active (Synytsya et al. 2017).

The extracts of different endophytic fungi, *A. nomius, A. oryzae, A. niger, A. terrus*, and *A. nidulans*, isolated from different tissues (leaf, stem, and root) of *Calotropis procera* (Apocynaceae), were evaluated for their antibacterial potential against nine bacterial strains. The tested extracts showed activity against all tested bacterial strains, with IZDs ranging from 10 to 17.3 mm and MICs ranging from 15.6 to 250 μg ml^−1^ (Rani et al. 2017).

Ten species of *Aspergillus* were isolated from soil samples obtained from two different Algerian regions namely: Laghouat and Teleghma. The ten species were identified as *A. fumigatus, A. niveus, A. wentii, A. fumigatiaffinis, A. quadrilineatus, A. nidulans, A. terreus, A. flavus, A. sclerotiorum*, and *A. niger*. Using disc diffusion method, the extracts of these fungi exerted activity against *S. aureus* (ATCC 25923), *B. subtilis* (ATCC 6633), and *E. coli* (ATCC 25922), with IZDs ranging from 7 to 31.7 mm, and the highest activity was by *A. fumigatus* against *S. aureus*, with IZD of 31.7 mm. The extracts of all species had no activity on *P. aeroginosa* (ATCC 9027), except the extracts of *A. niveus* and *A. wentii*, with IZDs 8 and 7 mm, respectively (Amina et al. 2017).

The ethyl acetate extract of *A. niger* isolated from the stilt roots of *Rhizophora apiculata* (Rhizophoraceae) along South Andaman coast, India, was assayed for bioactivity against five pathogenic bacterial strains: *S. aureus* (MTCC96), *M. luteus* (MTCC106), *P. aeruginosa* (MTCC326), *E. faecalis* (MTCC439), and *P. mirabilis* (MTCC1429) by disc diffusion method. Sensitivity of the strains varied from *P. mirabilis*, which was most sensitive followed by *P. aeruginosa, M. leuteus, S. aureus*, and *E. faecalis* in decreasing order, with IZDs ranged from 15 to 23 mm (Thorati and Mishra 2017).

## Conclusions

The rapid development in antibiotic resistance in bacteria has generated an increased demand for the development of novel therapies to treat bacterial infections. The present review focuses on antibacterial activity of *Aspergillus* spp. isolated from terrestrial environment from all over the world. *Aspergillus* spp. show antibacterial activity against pathogenic bacteria. According to literature, isolated compounds and extracts from *Aspergillus* spp. exhibit higher antibacterial activity against Gram-positive than Gram-negative bacteria. While several compounds exhibited higher IZDs or MICs than an antibiotic, some of them, such as the compound CJ-17,665, exhibited good activity against multi-drug resistant *S. aureus*, which is one of the most important current public health problem. Therefore, *Aspergillus* spp. may provide metabolites that may help treat infectious diseases that have increased resistance to current antibiotics, and could provide alternative medical treatment.
